# Supplementary figures and images for: Open reproducible scientometric research with Alexandria3k
Source: PLoS One. 2023 Nov 30;18(11):e0294946. doi: 10.1371/journal.pone.0294946 (PMC10688655; doi:10.1371/journal.pone.0294946)

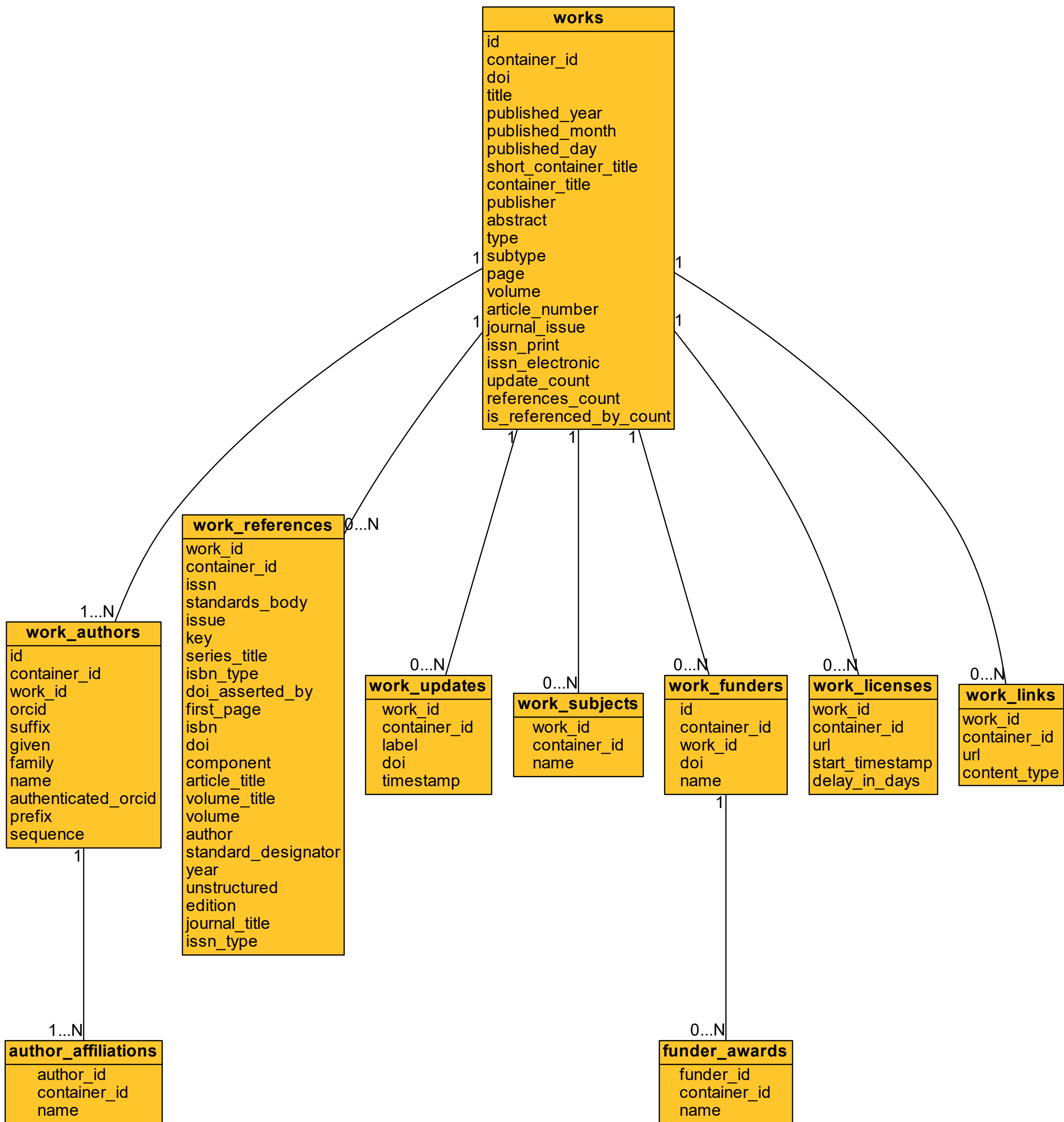

Supplement: S1 Fig — (PDF) [file pone.0294946.s001.pdf]

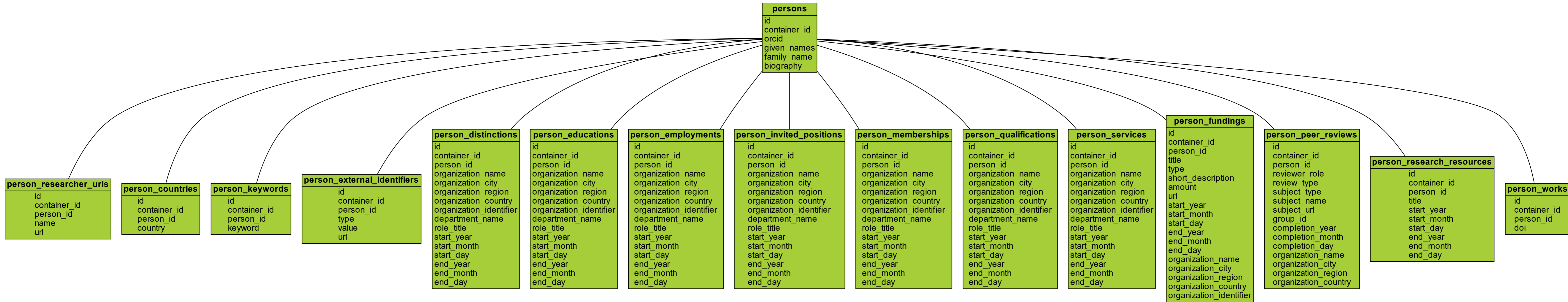

Supplement: S2 Fig — (PDF) [file pone.0294946.s002.pdf]

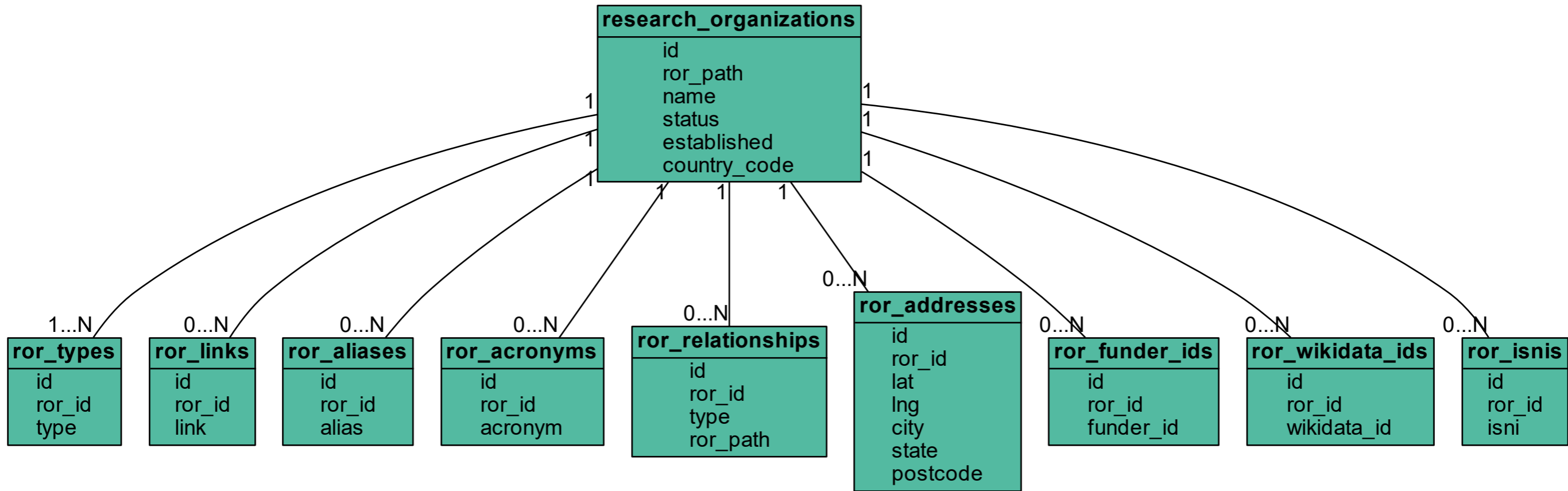

Supplement: S3 Fig — (PDF) [file pone.0294946.s003.pdf]
