# Supplementary material for: Open reproducible scientometric research with Alexandria3k
Source: PLoS One. 2023 Nov 30;18(11):e0294946. doi: 10.1371/journal.pone.0294946 (PMC10688655; doi:10.1371/journal.pone.0294946)
Supplement: S4 Fig — (PDF) [file pone.0294946.s004.pdf]

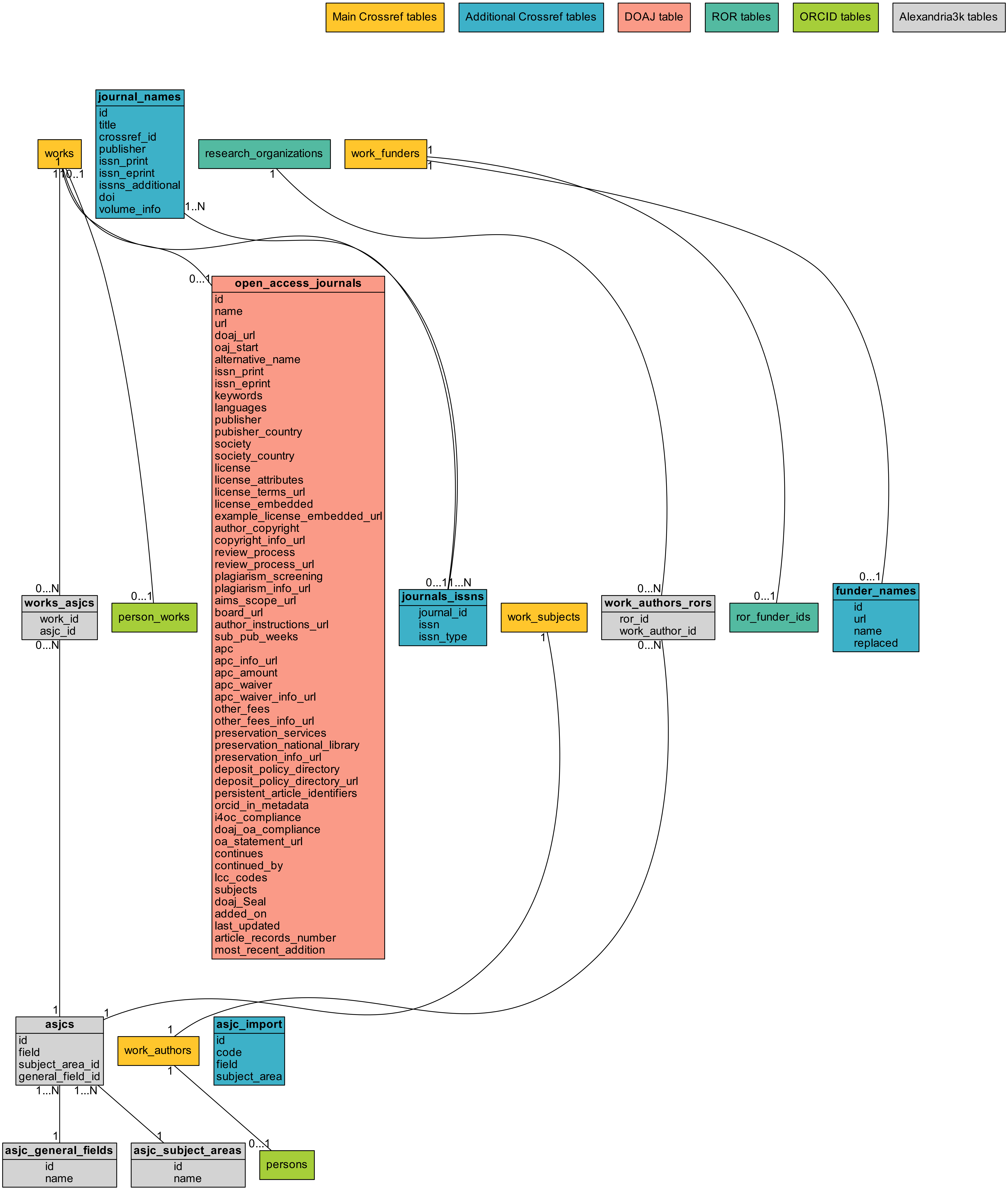

Main Crossref tables
Additional Crossref tables
DOAJ table
ROR tables
ORCID tables
Alexandria3k tables
journal_names
id
title
crossref_id
publisher
issn_print
issn_eprint
issns_additional
doi
volume_info
works
research_organizations
work_funders
open_access_journals
id
name
url
doaj_url
oaj_start
alternative_name
issn_print
issn_eprint
keywords
languages
publisher
pubisher_country
society
society_country
license
license_attributes
license_terms_url
license_embedded
example_license_embedded_url
author_copyright
copyright_info_url
review_process
review_process_url
plagiarism_screening
plagiarism_info_url
aims_scope_url
board_url
author_instructions_url
sub_pub_weeks
apc
apc_info_url
apc_amount
apc_waiver
apc_waiver_info_url
other_fees
other_fees_info_url
preservation_services
preservation_national_library
preservation_info_url
deposit_policy_directory
deposit_policy_directory_url
persistent_article_identifiers
orcid_in_metadata
i4oc_compliance
doaj_oa_compliance
oa_statement_url
continues
continued_by
lcc_codes
subjects
doaj_Seal
added_on
last_updated
article_records_number
most_recent_addition
works_asjcs
work_id
asjc_id
person_works
journals_issns
journal_id
issn
issn_type
work_subjects
work_authors_rors
ror_id
work_author_id
ror_funder_ids
funder_names
id
url
name
replaced
asjcs
id
field
subject_area_id
general_field_id
work_authors
asjc_import
id
code
field
subject_area
asjc_general_fields
id
name
asjc_subject_areas
id
name
persons
