# Supplementary material for: Open reproducible scientometric research with Alexandria3k
Source: PLoS One. 2023 Nov 30;18(11):e0294946. doi: 10.1371/journal.pone.0294946 (PMC10688655; doi:10.1371/journal.pone.0294946)
Supplement: Attachment — Submitted filename: response-to-reviewers.pdf [file pone.0294946.s005.pdf]

Date September 14th, 2023
Our reference alexandria3k e29d53d
Contact person Prof.dr.ir. Diomidis Spinellis
Web www.spinellis.gr
E-mail D.Spinellis@tudelft.nl
Subject Manuscript PONE-D-23-15521

TU Delft

Faculty of Electrical Engineering,
Mathematics and Computer Science
Department of Software Technology

Visiting address
Mekelweg 4
2628 CD Delft

Postal address
P.O. Box 5031
2600 GA Delft
The Netherlands

Article title: "Open reproducible scientometric research with Alexandria3k"

Dear colleagues,

Thank you for the review response inviting me to submit a revised version of the manuscript PONE-D-23-15521] - [EMID:c04888fb3ccc1ea6] that addresses the points raised during the review process. I wish to thank the Editor and the reviewers for their useful feedback and constructive comments. Please below the responses summarizing the changes made. Furthermore, I am submitting through the online system **two documents with tracked changes**, highlighting the changes made in response to each reviewer's comments.

Yours sincerely,

Diomidis Spinellis

Review Response

Journal requirements

When submitting your revision, we need you to address these additional requirements.
1. Please ensure that your manuscript meets PLOS ONE's style requirements, including those for file naming. The PLOS ONE style templates can be found at https://journals.plos.org/plosone/s/file?id=wjVg/PLOSOne_formatting_sample_main_body.pdf and https://journals.plos.org/plosone/s/file?id=ba62/PLOSOne_formatting_sample_title_authors_affiliations.pdf
2. Thank you for stating the following in the Competing Interests section:

“Google Summer of Code through the Open Technologies Alliance supported the work of a student developer on Alexandria3k. The author has declared that no competing interests exist.”
Please confirm that this does not alter your adherence to all PLOS ONE policies on sharing data and materials, by including the following statement: "This does not alter our adherence to PLOS ONE policies on sharing data and materials.” (as detailed online in our guide for authors http://journals.plos.org/plosone/s/competing-interests). If there are restrictions on sharing of data and/or materials, please state these. Please note that we cannot proceed with consideration of your article until this information has been declared.
Please include your updated Competing Interests statement in your cover letter; we will change the online submission form on your behalf.

The revised statement is included in the revised cover letter.

3. In your Data Availability statement, you have not specified where the minimal data set underlying the results described in your manuscript can be found. PLOS defines a study's minimal data set as the underlying data used to reach the conclusions drawn in the manuscript and any additional data required to replicate the reported study findings in their entirety. All PLOS journals require that the minimal data set be made fully available. For more information about our data policy, please see http://journals.plos.org/plosone/s/data-availability.
Upon re-submitting your revised manuscript, please upload your study’s minimal underlying data set as either Supporting Information files or to a stable, public repository and include the relevant URLs, DOIs, or accession numbers within your revised cover letter. For a list of acceptable repositories, please see http://journals.plos.org/plosone/s/data-availability#loc-recommended-repositories. Any potentially identifying patient information must be fully anonymized.
Important: If there are ethical or legal restrictions to sharing your data publicly, please explain these restrictions in detail. Please see our guidelines for more information on what we consider unacceptable restrictions to publicly sharing data: http://journals.plos.org/plosone/s/data-availability#loc-unacceptable-data-access-restrictions. Note that it is not acceptable for the authors to be the sole named individuals responsible for ensuring data access.
We will update your Data Availability statement to reflect the information you provide in your cover letter.

The original submission’s data availability statement included DOIs and URLs of the used data sets. It has now been revised in the submission system as follows to include only DOIs to stable public recommended repositories.

“A versioned release of the source code of Alexandria3k and the proof-of-concept examples presented in this study is available on Zenodo doi: 10.5281/zenodo.7953287, licensed under the GNU General Public License v3.0. A replication package with the results data and Python scripts used for the statistical analysis and charting of the reported example studies is also available on Zenodo doi: 10.5281/zenodo.8273279. Current versions of Alexandria3k are made available for installation through PyPi https://pypi.org/project/alexandria3k/ and for contributions, feature requests, and issue reporting on GitHub https://github.com/dspinellis/alexandria3k.

The data used in the example studies are available as follows.

Crossref Apr. 2022 Public Data File: doi:10.13003/83b2gq

ORCID Public Data File 2022 v. 4: doi:10.23640/07243.21220892.v4
ROR Data v1.17.1: doi:10.5281/zenodo.7448410
Open access journals: https://doaj.org/csv doi:10.5281/zenodo.8265889
Funders: https://doi.crossref.org/funderNames?mode=list
doi:10.5281/zenodo.8265889
Journals: http://ftp.crossref.org/titlelist/titleFile.csv doi:10.5281/zenodo.8265889

The calculated CD_5 index of works published in the period 1945-2016 dataset is available on Zenodo doi: 10.5281/zenodo.7679112. The 2021 Journal Impact Factor data used for assessing the numbers obtained by Alexandria3k are available from Clarivate through the InCites Journal Citation Reports service https://jcr.clarivate.com/."

The above data sets and the replication package are now also cited in the paper, as recommended by the journal's guidelines.

Comments to the Author

Reviewer #1:
This manuscript tackles the issue of reproducible scientometric studies. It introduces the Alexandria3k Python software package that is showcased on a case study of COVID-19 research.

The paper is very well organised and meticulously written. It manages to motivate the need for reproducible research in scientometrics and introduces the features of Alexandria3k using laymen terms while providing technical details that will inform specialists of database design. I commend the author for stressing the limitations of his case study relying on open yet incomplete data, in the Discussion section.

I recommend 'Minor Accept' and welcome the author to consider the following comments to revise the manuscript:

** C1. The title seems not specific enough*

I believe the title should better reflect the contribution by referring to scientometrics and Alexandria3k. A tentative title:

Open reproducible scientometric research with Alexandria3k

The title has been revised as recommended.

** C2. Mention of downstream software for bibliometric data analysis and visualisation*

I kept thinking of bibliometrix (https://www.bibliometrix.org) while reading the submission. I believe readers would benefit from a paragraph explaining how the data harvested by Alexandria3k could feed bibliometrix for further data foraging.

Aria, M. & Cuccurullo, C. (2017). bibliometrix: An R-tool for comprehensive science mapping analysis, Journal of Informetrics, 11(4), pp 959-975, Elsevier, DOI: 10.1016/j.joi.2017.08.007

The following paragraph has been added at the end of the Contents, structure, and use" section: "The analysis of the extracted data can be performed either directly with SQL queries and ad hoc code, as shown in the next section, or by utilizing specialized software. For example, once \name\ has populated a database, its data can processed in R[…] using the bibliometrix tool for science mapping analysis[…]. This can be done by creating R data frames or tibbles (enhanced data frames provided by the tidyverse package) from data brought into R through its DBI and RSQLite packages."

** C3. OpenAlex as a potential source*

The author cites OpenAlex on page 4, mentioning that pySciSci sources from OpenAlex. I understand that OpenAlex aggregates and cleanses data from various bibliographic data providers, such as Crossref. Would if make sense to feed Alexadria3k with the OpenAlex snapshot (https://docs.openalex.org/download-all-data/openalex-snapshot)? Could the author consider discussing this so that authors learn about the pros and cons of this data ingestion strategy?

The idea of adding OpenAlex (and other) data to Alexandria3k is now further discussed in the conclusions: "Apart from making Alexandria3k available as open-source software we also structured it and intend to run it as an open-source project, accepting and integrating contributions of additional open-data sources (e.g. MEDLINE/PubMed, patent metadata, OpenAlex [36], algorithms (e.g. publication topic classification; author name and affiliations disambiguation; citation matching), and example studies. Some of the data sources, such as PubMed and OpenAlex, overlap with Crossref, but can be useful for the additional metadata they provide, such as the MEDLINE medical subject headings (MeSH) and the OpenAlex concepts. A practical approach for integrating such data may be to create data sources that only import the metadata and link them with the works table using the DOI."

** C4. Clarification regarding COVID-19 publications*

On page 12, the authors mentions COVID-19 publications. I wondered about the share of preprints vs journal article in the harvested dataset, since preprints were so instrumental to the COVID-19 research.

The following paragraph now appears in the corresponding section: "Finally, we examined the heightened role of preprints in COVID-19 publications […]. In the COVID data set from the 491 945 published works, 48 211 (9.8%) were identified as preprints. Contrast this with all the works published in the same period where from the 16 369 968 published works, 572 200 (3.5%) were identified as preprints." The data and scripts used to obtain them have been added in the replication package.

** C5. Potential use of multithreading*

p18: "the evaluation is evaluated sequentially on each Crossref container" -> could the author discuss the pros and cons of multithreading to perform parallel data loading?

This is a good point. In response, the author tried some approaches to perform parallel data loading, but without success. A sentence at the end of the paragraph now reads: "Unfortunately, multithreading cannot be easily used to further improve performance because both Python and SQLite make it difficult to obtain the required concurrency."

** Misc*

- p13: "with a p-value 3 × 10^{−93}" (and elsewhere) -> p < 0.001, see the submission guidelines (https://journals.plos.org/plosone/s/submission-guidelines.#loc-statistical-reporting) and comments on social media (https://twitter.com/hippopedoid/status/1673806261895266306/retweets/with_comments).

Six instances of this issue were fixed.

- p17: "MondGB" -> MongoDB

Fixed.

- p21: "DOI as a key" -> since DOI resolution is case insensitive, it might be worth specifying that all DOIs where lowercased prior to any matching process -- if that's the case indeed.

The paragraph now ends as follows: "(To facilitate the required case-insensitive DOI matching, \name\ converts internally all DOIs into lowercase.)"

- p21: "by extracting the starting and ending page number [...] We excluded from the data records with...". Did the author exclude records featuring pageStart > pageEnd?

Yes, such records were excluded. The description now reads "We excluded from the data records with a starting page lower or equal to the ending page or …"

- p25: "the true number of authors" in ORCID. The author might appreciate reading:

Teixeira da Silva, J. A. (2021). Abuse of ORCID's weaknesses by authors who use paper mills. In Scientometrics (Vol. 126, Issue 7, pp. 6119–6125). https://doi.org/10.1007/s11192-021-03996-x

The author indeed appreciated reading the paper. It documents another sad manifestation of the rule "When a measure becomes a target, it ceases to be a good measure." The corresponding paragraph now ends with the following sentence: "Note that the employed method's accuracy is still vulnerable to ghost and empty ORCIDs that seem to be created by paper mills […]."

- As a complement to [88] on Chinese surnames, the author might appreciate reading:

Youtie, J., Carley, S., Porter, A. L., & Shapira, P. (2017). Tracking researchers and their outputs: new insights from ORCIDs. In Scientometrics (Vol. 113, Issue 1, pp. 437–453). https://doi.org/10.1007/s11192-017-2473-0

True, the issues identified in the study should be acknowledged. The corresponding paragraph now ends as follows: "The different percentages at the two periods' ends indicate that further controls for this change would be required, e.g. by measuring name clashes through ORCIDs while also considering variations in their adoption […]."

- The author could consider replacing [31] by:

Hendricks, G., Tkaczyk, D., Lin, J., & Feeney, P. (2020). Crossref: The sustainable source of community-owned scholarly metadata. In Quantitative Science Studies (Vol. 1, Issue 1, pp. 414–427). https://doi.org/10.1162/qss_a_00022

The proposed is indeed a more recent and better reference in terms of quality and credit. It has replaced the original one.

Reviewer #2:
The paper presents Alexandria3k, a Python package that can populate relational databases with data from several open publication metadata datasets, including CrossRef, ORCID, and ROR. Alexandria3k enables scientists and science analysts to perform reproducible quantitative studies of science without recurring to the usual commercial products, such as Web of Science or Scopus.

Even if I am sympathetic with this project and I think Alexandria3k might be a useful tool to add to the toolbox of science analysts, I must confess that I am a bit pessimistic about its capacity to realistically compete with commercial products that can rely on massive financial resources and do not have many of the limitations of open-access datasets such as CrossRef. That said, Alexandria3k is in my opinion technically sound and can be useful, especially to members of the scientific community that cannot access commercial databases.

My main concern is the overall structure of the paper. In its present form, it is very long and presents too much material to the readers. I would suggest to the Author(s) to shorten the paper significantly and reorganize its content in order to enhance the clarity of the presentation. For instance, the paper begins in medias res, so to say, by presenting a result obtained with Alexandria3k, even before the tool is properly introduced and the problems it aims to resolve presented. In the current version of the Introduction, the readers find themselves in the middle of numerous figures and statistics about the raise of science studies without a context, leaving them confused. I would rather suggest the Author(s) to start the paper with a classic overview of the problem Alexandria3k is meant to resolve and with a description of the state of the art around the problem (i.e., expand the section that starts around row 23).

The introduction has been restructured to follow this recommendation. The evolution of synthesis studies has been integrated into the application example on the evolution of scientific publishing. Furthermore, the introduction, in addition to the already cited online data sources and analysis platforms, now also includes references to related software systems with a clarification of Alexandria3k's advantage.

Moreover, I would suggest the Author(s) to reduce the number of proof-of-concept studies of Alexandria3k and to significantly shorten the Methods section: I would select three or four studies and present them in separate paragraphs, with research question, methods, and results of each of them clearly explained, avoiding useless technical minutiae. Further studies can be left to Supplementary Materials, added to the supporting information of the package or even presented in a separate paper.

The "Cross-disciplinary research"proof-of-concept study was entirely removed (together with the associated description in the Methods section), and is now distributed with the system's source code. The remaining examples were consolidated into three studies: Evolution of scientific publising, COVID-19 research, and

Productivity metrics. Less useful technical details were eliminated from the Methods section. These changes reduced the manuscript length by three pages over the originally submitted one. Methods were not integrated into each study, because a lack of a one-to-one matching between the two would result in an overly complex document structure and internal cross-referencing.

After these changes in the structure of the paper, in my view it can proceed to publication.

Other changes

While editing the document for resubmission, the following minor additional changes were made.

- A space character was added before the GHz unit in the Table 3 footnotes.
- A reference was added to the article by Sing Chawla (2022) regarding citation availability.
- A fix in the computations' elapsed time calculation resulted in an update of the corresponding figures presented in Table 3.
- Performance figures for the CD_5 index calculation were added.
